# The IncRNA SCIRT Promotes the Proliferative, Migratory, and Invasive Properties of Cervical Cancer Cells by Upregulating MMP-2/-9

**DOI:** 10.1155/2022/3448224

**Published:** 2022-08-08

**Authors:** Chunfeng Guan, Beibei Wang, Qixiu Dong

**Affiliations:** ^1^Prenatal Diagnosis in Obstetrics and Gynecology, The Second Hospital of Anhui Medical University, Hefei, Anhui, China; ^2^Department of Obstetrics and Gynecology, Yancheng TCM Hospital Affiliated to Nanjing University of Chinese Medicine, Yancheng, Jiangsu, China

## Abstract

**Objective:**

The morbidity and mortality of cervical cancer (CC) rank the fourth-most common among cancers in females, seriously threatening women's health and affecting their quality of life. However, the molecular mechanism of CC development remains poorly understood. This study investigates the role of lncRNA SCIRT in the development of CC.

**Methods:**

The expression profile of long noncoding RNA stem cell inhibitory RNA transcript (lncRNA SCIRT) in CC (*n* = 34), tumor-adjacent tissue, and CC cell culture was determined through fluorescence quantitative PCR. The knockdown /overexpressed lncRNA SCIRT vectors were constructed and transfected into cells, and the effects of knockdown or overexpression of lncRNA SCIRT on the proliferative, invasive, and migratory properties of CC cells were determined through Cell Counting Kit-8 (CCK-8), colony forming, and Transwell experiments. Western blot was employed to determine the knockdown/overexpression efficiency of SCIRT and its role on the expression of proteins (e-cadherin, n-cadherin, vimentin, matrix metalloproteinase (MMP)-9 and MMP-2) in CC cells. Finally, SCIRT knockdown on the proliferative ability for CC cells was determined through tumorigenic experiment in nude mice.

**Results:**

LncRNA SCIRT was highly expressed in CC tissues and cells, and significantly linked with clinical/pathology-based characteristics of patients, including Federation Internationale of Gynecologie and Obstetrigue (FIGO) stage, tumor dimensions, and lymph-node metastasis. SCIRT knockdown markedly reduced CC proliferative, colony forming, and invasive properties, while overexpressing SCIRT promoted the proliferative and invasive properties of CC. Western blotting analysis demonstrated that SCIRT knockdown upregulated e-cadherin and downregulated n-cadherin, vimentin, MMP-9, and MMP-2. Meanwhile, overexpressing SCIRT of lncRNA SCIRT had the opposite effect. Tumorigenic experiment showed that SCIRT knockdown could markedly reduce CC proliferative property the nude mouse.

**Conclusion:**

LncRNA SCIRT was highly expressed in CC clinical cases. Knockdown/overexpressing SCIRT affected CC proliferative/invasive properties. Hence, lncRNA SCIRT is a promising drug-target and a new biological diagnostic molecule for CC patients.

## 1. Introduction

Cervical cancer (CC) has the fourth-highest global morbidity/mortality rates in women worldwide [1]. At present, screening programs have been developed for CC screening. Most CC can be diagnosed at early stage, which greatly improves the survival rate of patients [2]. CC morbidity/mortality statistics have declined with the increased popularization of vaccines, but CC is still a life-threatening disease according to the World Health Organization (WHO) [3, 4]. Late-stage recurrence and metastasis are the main factors affecting the prognosis of CC, and late-stage CC is prone to poor efficacy and poor prognosis, with the median survival time of late-stage patients being only 16.8 months [5, 6]. Therefore, early detection and early treatment is pivotal for improving prognostic odds in CC cases, and further molecular research to find new biological targets for promoting early-diagnosis and targeted CC therapy is needed.

Recently, long noncoding RNAs (lncRNAs) have gained increased attention due to their roles in human diseases including cancer [7, 8]. LncRNAs are >200 nucleotide-long non-protein-coding RNA molecules and are implicated in multiple physiological activities such as chromosomal re-modeling, epigenomic regulation, transcription-based and post-transcriptional modifications, together with cell proliferative/differentiating properties. LncRNA-driven regulation on body physiological functions is mainly realized by epigenetics, transcription, and post-transcription [8, 9]. Multiple investigations have shown that lncRNAs are closely related to CC development and progression. For example, lncRNA metastasis-associated lung adenocarcinoma transcript 1 (MALAT1) was found to be expressed in CC cells, and absent in healthy cervical specimens, suggesting that lncRNA MALAT1 plays important roles in CC occurrence [10]. Further studies showed that MALAT1 promotes CC proliferative and migratory properties through upregulating cyclin D1, cyclin *E*, and cyclin-dependent-kinase-6 (CDK6) [11]. Furthermore, it has been found that human papillomavirus (HPV)16 E6/E7 are implicated in MALAT1 upregulation in CC, indicating HPV to be a valorized parameter driving MALAT1 triggering CC [12]. Down-regulation of MALAT1 expression could markedly reduce proliferative property of HeLa/Caski cells, promote apoptotic rate, inhibit their invasive ability. Down-regulation of MALAT1 prevents CC advancement through downregulating miR-429 [13]. LncRNA Cervical carcinoma high expressed 1 (CCHE1) expression is significantly increased in CC in comparison with healthy tissue. Through function gain/function loss experiments, it was found CCHE1 overexpression exacerbated CC proliferative property. Additional mechanistic investigations demonstrated the effect of CCHE1 on the proliferative property of CC depends upon proliferating cell nuclear antigen (PCNA) upregulation. CCHE1 can bind to PCNA mRNA to upregulate PCNA, thus exacerbating proliferative property of CC cells [14, 15]. In terms of clinical/pathology-based features, CCHE1 upregulation remains highly linked with advanced Federation Internationale of Gynecologie and Obstetrigue (FIGO), large tumor diameter, lymph node metastasis, and positive HPV [13]. The above results suggest that lncRNAs are important molecules involved in CC tumorigenesis and progression, and can contribute to novel concepts for theragnostic measures against CC.

LncRNA Stem Cell Inhibitory RNA Transcript (lncRNA SCIRT) is a conservated lncRNA with important functions. This gene affects the occurrence and progression of breast cancer through controlling signal transduction pathways related to tumor cell cycle and self-renewal [16, 17]. In addition, lncRNA SCIRT promotes cancer progression through exosome metastasis in lung cancer tissues, which can contribute to novel targets of lung tumor theragnostics [18]. The SCIRT expression and its functional roles in CC remain unknown. This project aimed to investigate the properties of SCIRT in CC tissue and cell lines. The preliminary molecular mechanism was also explored. This study will provide molecular targets aimed at CC early-diagnostics and therapeutics.

## 2. Materials and Methods

### 2.1. Specimen Collection

The tumor tissues/tumor-adjacent tissues of 34 clinical cases having upper CC were collected after obtaining the approval through the Ethics Committee of the Second Affiliated Hospital of Anhui Medical University and informed-consent signed by patients or their families. The age range of enrolled patients was 36–69 years. The tissue samples of all patients were confirmed by 2 pathologists as CC. All patients were first surgery patients. Patients did not receive any preoperative radiotherapy or other antitumor treatment. The clinical data such as gender, age, pathological type, tumor dimension, differentiation level, lymph node metastases, and recurrence were completely recorded. All patients were followed up by out-patient visit and telephone call, and death or recurrence was regarded as the outcome event.

### 2.2. Cell Culture

CC cells HeLa, SiHa, C-33A, and HT-3 and healthy cervical epithelial immortalized H8 cells were obtained from the Cell Center of Chinese Academy of Medical Sciences. Cells were grown in DMEM (+10% fetal bovine serum) containing 100 U/ml penicillin/100 mg/mL streptomycin, and incubated at 5% CO_2_/37°C/saturated humidity. DMEM was replaced at 2-3-day intervals.

### 2.3. Fluorescence Quantitative PCR Assay (RT-qPCR)

Trizol method was adopted to extract total RNA from tissues/cellular cells, then Nanodrop2000 was employed to determine the concentration of nucleic acid. The total RNA was stored at -80°C for later use. ABI 7500 was employed for RT-qPCR detection, while two-step technique was adopted for expansion. The reaction conditions consisted of: pre-denaturation at 95°C for 30 s; 95.0°C for 5 s/60.0°C for 30 s × 40 cycles, 72°C for 30 s. After the reaction, the dissolution curve was generated by the built-in software of the PCR Amplifier. Relative expression result of the lncRNA/gene of interest in each group was analyzed using the 2^−ΔΔCt^ technique. ΔΔCt = ΔCt (experimental group) -ΔCt (control group), and ΔCt = Ct (target gene) -ΔCt (*β*-actin). Ct reflected number of amplification cycles required for fluorescence level to reach threshold. Primer sequences of lncRNA SCIRT and glyceraldehyde-3-phosphate dehydrogenase (GAPDH) were the same as those in reference [19].

### 2.4. Transfection Experiment

CC cells were seeded in 6-well plates. Once cell confluence reached about 90%, the cells in the 6-well plates were cultured with serum-free medium. After adding 8–10 MOI of virus, the cells were incubated at 37°C for two hours, with gentle shaking every 15 min. Then 2 ml medium was added, and the 6-well plates were carefully shaken to make the mixture fully mixed. The plates were consequently incubated at 37°C and 5% CO_2_ for 48 h. Subsequently, the original supernatant of the medium was discarded, and full-blown DMEM was introduced for continuing culture in the incubator.

### 2.5. Cell Viability Detection by Cell Counting Kit-8 (CCK-8) Assay

CCK-8 was employed to detect the cell proliferative activity. First, cells having good growth condition were harvested. After pouring out the original medium, cells were twice-washed with pre-cooled phosphate buffer saline (PBS) buffer. Then, cells were exposed to 0.25% trypsin, rendered into single-cell suspensions. Post-cellular population quantification, cell density was adjusted, and inoculated into a 96-well plate at 5 × 10^3^ cells/well. Five replicates were designated for individual wells, and each well was added with 100 *μ*L medium. The plate was gently shaken to even the cells. Consequently, cells were incubated (37°C/5%CO_2)_. Medium was replaced during 0 h, 24 h, 48 h, and 72 h after transfection, and 10 *μ*L CCK-8 reagent was introduced into all wells. This procedure was performed according to the manufacturer's protocols, and such cells were incubated at 37°C for 2 h in the dark. Finally, the 96 well plate was put into a microplate reader for obtaining optical density (OD) at 450 nm. The average value of OD was calculated, and the cell growth curve of each group was plotted according to the results.

### 2.6. Colony Forming Assay

First, cells in good growth condition were collected. After pouring out the original medium, the cells were double-washed using pre-cooled PBS buffer. Then, cells were exposed to 0.25% trypsin, rendered into single-cell suspensions. Post-cellular population quantification, cell density was adjusted, and inoculated into 6-well plates (700 cells/well). The 6-well plates were pre-washed with pre-cooled PBS buffer. The plate was gently shaken to even the cells and consequently incubated as previously described. Cell growth was observed regularly, and the medium was replaced on time. The culture was stopped when visible cell colonies appear (about 14 days). After discarding the original medium, the cells were double-washed using PBS buffer, fixated using 2 mL paraformaldehyde for 15 minutes, and stained with 2 ml 1% crystal violet for 10 minutes. The 6-well plates were PBS-rinsed, dried at room temperature, and observed under a microscope. The images of colonies were collected for counting analysis (monoclonals with cells ≥50).

### 2.7. Transwell Assay on Cell Migration and Invasion

1. Cell migration: First, cells in good growth condition were collected. Then, cells were exposed to 0.25% trypsin, rendered into single-cell suspensions. Cell number quantification, cell density was adjusted to 2 × 10^6^ cells/mL using serum-free Dulbecco's modified eagle medium (DMEM). Next, 100 *μ*L cellular aliquots were taken and introduced into the upper-chamber of Tranwell, while 500 *μ*L DMEM +15% fetal bovine serum were introduced in lower-chamber. Culture was stopped after 36 h. The cells in the upper compartment were rinsed with PBS and removed with cotton swabs. The migrated cells were fixed with 700 *μ*L methanol for 20 minutes. After fixation, the cells were washed 3 times with PBS buffer solution, and 0.1% crystal violet dye was added for staining for about 10 min. Consequently, the cells were monitored through inverted microscopy, where quantity of cells stained by crystal violet in 5 fields was calculated.
2. Cell invasion: First, 50*μ* L pre-cooled Matrigel gel in 4°C refrigerator was coated over upper chamber of the Transwell Then, the chamber was balanced in a 4°C refrigerator for 30 min. The subsequent steps were the same as the cell migration experiment.

### 2.8. Western Blotting

First, adherent cells in a good growth condition were collected. After pouring out the original medium, the cells were double-rinsed using pre-cooled PBS. Then, an appropriate amount of RIPA (containing 1% phenylmethanesulfonyl fluoride (PMSF)) was added to the cell culture flask, and the lysis buffer was evenly distributed by gently shaking. The flask was placed on a 4°C shaker for lysis for 30 minutes. The supernatant was harvested through centrifugation, with the level of total protein samples was determined using the Bradford method. Proteins were subjected to electrophoresis with sodium dodecyl sulfate polyacrylamide gel electrophoresis (SDS-PAGE) and then transferred onto a poly(vinylidene fluoride) (PVDF) membrane that was blockaded by 5% skimmed milk-powder at an ambient temperature for 120 minutes and placed into incubation with 5% albumin from bovine serum (BSA) diluted 1°C antibodies e-cadherin (1 : 900), n-cadherin (1 : 1000), vimentin (1 : 800), metalloproteinase (MMP)-9 (1 : 700), and MMP-2 (1 : 700) at 4°C overnight. *β*-actin (1 : 1000) was employed as an internal parameter. The PVDF membrane was TBST (Tris-Buffered, Saline and Tween) buffer-rinsed and treated with a 2°C antibody diluted using 5% BSA (1 : 5000) to be placed into incubation at room temperature for 90 minutes. PVDF membrane was rinsed with TBST rinsed for 4 times, five minutes/rinse. With the protein surface of the membrane kept facing down, the membrane was placed in efficient chemiluminescence (ECL) reagent and incubated for 10 min without light. Then, the film was exposed and developed in the darkroom, and the photos were taken.

### 2.9. Tumorigenesis in Nude Mouse

Eight BALB/c female nude mice (4-5 week-old) were purchased from the Laboratory Animal Center of Shanghai, Academy of Science Chinese (Shanghai, China) and used to establish subcutaneous grafts. All the nude mice were house in a SPF laboratory animal room (22 ± 2°C with 50% ± 10% relative humidity). Unlimited drinking water and food was provided. This animal experiment was accepted by the Animal Ethics Committee of our institute. Cells were transfected in line with the above experimental steps. The cell density was adjusted to 1 × 10^7^ cells/mL using serum-free medium DMEM. BALB/c nude mice were segregated as sh-Control group and sh-SCTR group with 4 mice per group. Then, the nude mice were routinely disinfected in a sterile room and fixed after anesthesia. Cell suspension of 0.1 ml (about 1 × 10^6^ cells) was inoculated into the subcutaneous area of the back of the mice at a constant speed. Before inoculation, the back skin was disinfected with 75% alcohol. After inoculation, the nude mice were given routine feeding, and the general condition of the mice, including diet, spirit, body weight and tumor forming status, was observed regularly. The long-diameter (a) together with short-diameter (b) for xenograft tumor were measured once a day. The volume of the xenograft tumor was determined in line with:

(1) $$\begin{array}{c} \text{tumor volume}\left(\text{VT}\right)=\frac{ab^{2}}{2}, \end{array}$$

while growth curve for xenograft tumor of the mice was plotted based over the tumor volume change.

### 2.10. Statistical Analysis

SPSS 22.0® was employed in all analyses, and GraphPad Prism 7.0® software was employed for diagraph evaluation. All measurement datasets were expressed as mean ± standard deviation (*x* ± *s*). One-way ANOVA was employed for comparing between several groups, and *t*-test was employed for comparing between two groups. *P* < 0.05 was deemed to confer statistical significance.

## 3. Results

### 3.1. LncRNA SCIRT Is Highly Expressed in CC Tissues and Cells

**Figure 1:**
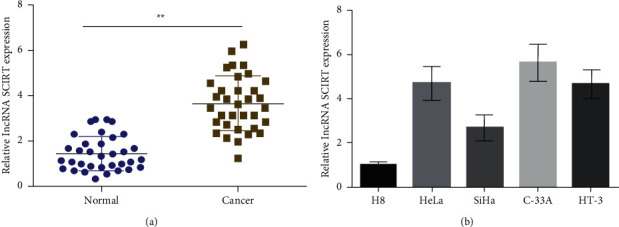
LncRNA SCIRT is upregulated in CC tissues and cells. (a) RT-qPCR detects SCIRT expression in 34 CC biopsies and adjacent tissues. (b) RT-qPCR detects SCIRT expression in CC cells and H8 cells. ^*∗∗*^*p* < 0.01.

First, quantitative PCR was employed for determining SCIRT expression in 34 CC and 34 tumor-adjacent tissues. The results showed that SCIRT was upregulated in CC (Figure 1(a)). Based on this data, 34 CC tissue samples were divided into high-expression group and low-expression group, and the association of SCIRT expression level and clinicopathological features in CC patients was analyzed. The results showed that upregulated SCIRT was highly linked to patients' clinicopathological characteristics, such as FIGO stage, tumor dimension and lymph node metastasis (Table 1). Besides, SCIRT expression in CC cells (HeLa, SiHa, C-33A and HT-3 cells), and H8, were determined through fluorescence quantitative PCR. The results showed that, in comparison to healthy cervical cells (H8), CC SCIRT level was considerably upregulated, with the highest expression level in C-33A cells. Meanwhile, SCIRT was downregulated in SiHa cells (Figure 1(b)). Therefore, C-33A and SiHa cells were chosen for additional investigations and molecular mechanism studies.

### 3.2. Effect of lncRNA SCIRT on CC Cell Proliferative Property

**Figure 2:**
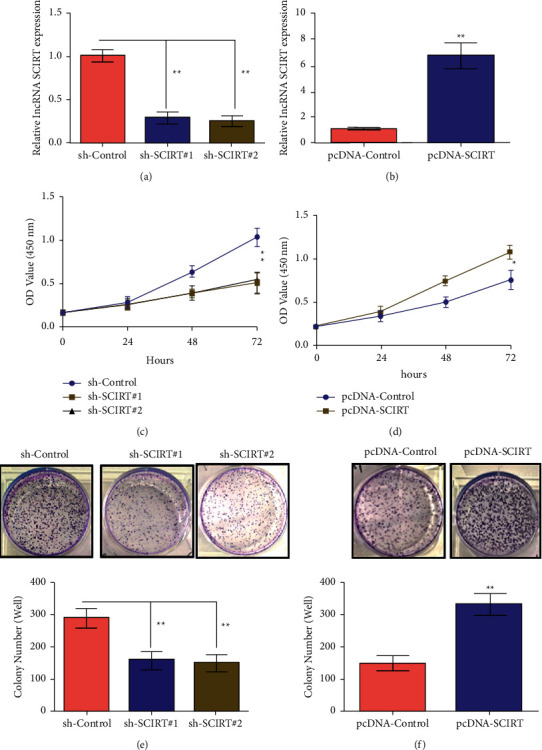
Knockdown or overexpressing SCIRT effects on the proliferative ability of CC cells. (a, b) Fluorescence quantitative PCR detects the expression of lncRNA SCIRT in CC cells (C-33A, SiHa) after knockdown or overexpressing SCIRT of lncRNA SCIRT; (c, d) CCK-8 detects the effect of knockdown or overexpressing SCIRT of LncRNA SCIRT over proliferation in C-33A and SiHa cells; (e, f) Colony forming assay detects the effect of knockdown or overexpressing SCIRT of LncRNA SCIRT on the colony forming ability of C-33A and SiHa cells. ^*∗*^*P* < 0.05, ^*∗∗*^*p* < 0.01.

Lentiviral vectors (sh-SCIRT#1, sh-SCIRT#2) and blank control vectors (sh-Control) targeting lncRNA SCIRT sequence were constructed, and transfected into C-33A cells. SCIRT expression in C-33A cells was determined through RT-qPCR. It was demonstrated that transfection of lentiviral vector (sh-SCIRT#1, sh-SCIRT#2) targeting SCIRT sequence significantly downregulated SCIRT in CC cells (Figure 2(a)). Meanwhile, lncRNA SCIRT overexpressing SCIRT vector (pcDNA-SCIRT)/control vector (pcDNA-Control) were also developed and transfected into SiHa cells. SCIRT expression level in SiHa cells was also determined through RT-qPCR. It was found that transfection of SCIRT overexpressing SCIRT vector (pcDNA-SCIRT) could significantly upregulate SCIRT in CC cells (Figure 2(b)). Furthermore, CCK-8 was employed for detecting SCIRT dysregulations (following knockdown or overexpressing SCIRT) over proliferative ability of CC cells. The results showed that SCIRT knockdown (sh-SCIRT#1, sh-SCIRT#2) markedly reduced proliferative property of CC C-33A cells (Figure 2(c)). In SiHa cells, compared with blank control vector (pcDNA-Control), transfection of lncRNA SCIRT overexpressing SCIRT vector (pcDNA-SCIRT) could significantly facilitate the proliferative ability of CC cells (Figure 2(d)). According to the colony forming assay, compared with blank control vector (sh-Control), SCIRT knockdown (sh-SCIRT#1, sh-SCIRT#2) significantly suppressed CC colony formation in C-33A cells (Figure 2(e)). In SiHa cells, transfection of lncRNA SCIRT overexpressing SCIRT vector (pcDNA-Control) significantly improved CC colony forming capacity compared with blank control vector (pcDNA-SCIRT) (Figure 2(f)). These results indicated that lncRNA SCIRT could significantly influence CC proliferation.

### 3.3. SCIRT Affects the Invasive and Migratory Ability of CC

**Figure 3:**
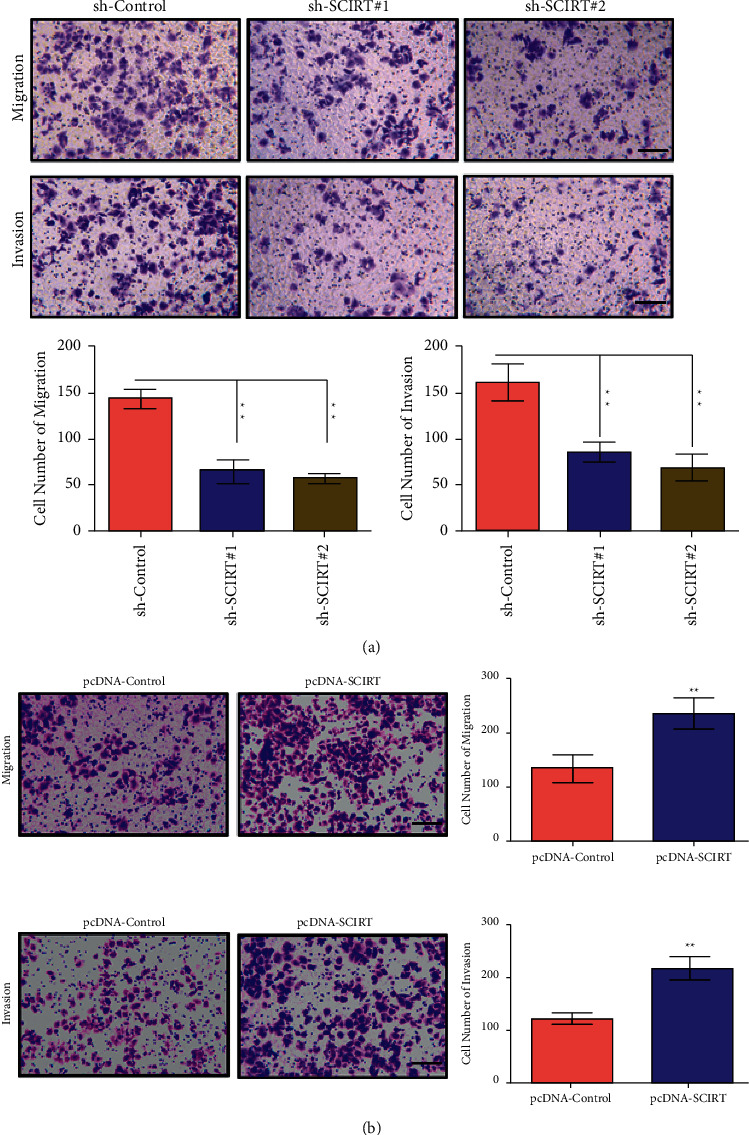
Transwell assay detects the effects of lncRNA SCIRT on the invasive and migratory ability of CC cells. (a) The effect of knockdown lncRNA SCIRT on cell migratory and invasive ability in C-33A cells. (b) The effect of overexpressing SCIRT of lncRNA SCIRT on cell migratory and invasive ability in SiHa cells. ^*∗∗*^*p* < 0.01, scale bar = 100 *μ*m.

Transwell migratory and invasive experiments were performed after knockdown and overexpressing SCIRT in C-33A and SiHa cells. The cell migratory experiment results showed that SCIRT knockdown (sh-SCIRT#1, sh-SCIRT#2) could significantly inhibit cell invasive and migratory ability in C-33A cells (Figure 3(a)). In SiHa cells, compared with blank control vector (pcDNA-Control), transfection of lncRNA SCIRT overexpressing SCIRT vector (pcDNA-SCIRT) can certainly promote CC invasive and migratory properties (Figure 3(b)). These results indicated that SCIRT affects the invasive and migratory properties of CC cells.

### 3.4. Effects of LncRNA SCIRT on the Expression of Invasion and Migration-Related Proteins in CC

**Figure 4:**
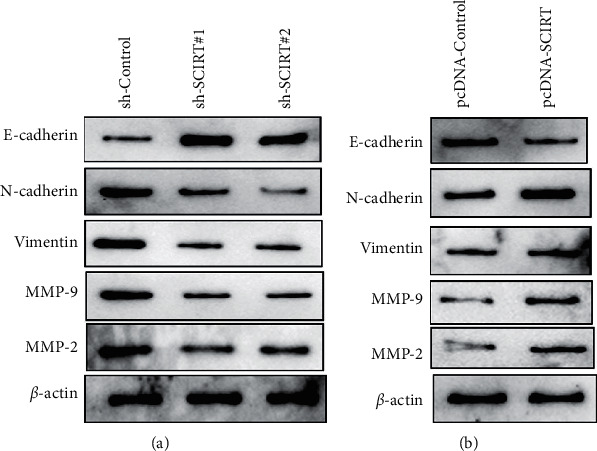
Western blotting analysis on the effects of knockdown or overexpressing SCIRT of lncRNA SCIRT on the expression of invasion and migration-related proteins in CC.

The epithelial-mesenchymal transition (EMT) is an important step for tumor cells to acquire the ability of invasion and metastasis. Western blotting was adopted for detecting EMT-linked proteins in each group. The results showed that compared with blank control vector (sh-Control), lncRNA SCIRT knockdown (sh-SCIRT#1, sh-SCIRT#2) markedly downregulated n-cadherin, vimentin, MM-9, and MMP-2, while upregulating e-cadherin in C-33A cells (Figure 4). In SiHa cells, compared with the blank Control vector (pcDNA-Control), transfection of lncRNA SCIRT overexpressing SCIRT vector (pcDNA-SCIRT) significantly upregulated n-cadherin, vimentin, MM-9, and MMP-2, while downregulating e-cadherin (Figure 4). These results highlight that SCIRT can significantly affect the invasion/migration associated protein expression in CC cells.

### 3.5. SCIRT Knockdown Considerably Inhibits the Proliferative Ability of CC in Nude Mouse

**Figure 5:**
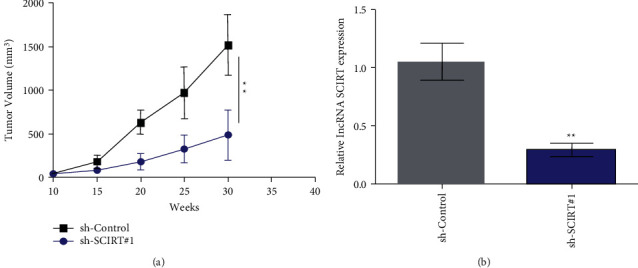
LncRNA SCIRT knockdown could markedly reduce proliferative ability of CC cells in nude mouse. (a) SCIRT knockdown affects CC growth in nude mouse; (b) Fluorescence quantitative PCR detects the expression of lncRNA SCIRT in the xenograft tumors. ^*∗∗*^*p* < 0.01.

In order to additionally validate SCIRT knockdown effects on the CC cell proliferative ability in vivo, tumorigenic experiment was performed in nude mouse. The results demonstrated that xenograft tumor volumes in SCIRT knockdown (sh-SCIRT#1) group was significantly reduced (Figure 5(a)). Fluorescence quantitative PCR results demonstrated that the SCIRT expression in xenograft tissues of SCIRT knockdown (sh-SCIRT#1) group was significantly reduced (Figure 5(b)). Such dataset outcomes suggested SCIRT knockdown could markedly reduce CC proliferative ability in nude mouse.

## 4. Discussion

A significant amount of basic and clinical studies have shown that development of CC is a complex process. In addition to human papillomavirus (HPV) infection, activation of oncogene or inactivation of suppressor genes also play pivotal roles in cancer advancement [19, 20]. Accumulating evidence indicates that some lncRNAs are abnormally expressed in CC and can regulate the proliferative, migratory, and apoptosis of CC through various pathways [21]. Therefore, identifying lncRNAs and clarifying their functions and effects are of great importance at multiple levels. The results of the present study proved that lncRNA SCIRT was highly expressed in CC tissues and cells and was significantly correlated with the clinicopathological characteristics of patients, such as FIGO stage, tumor dimension, and lymph node metastasis. SCIRT knockdown could significantly inhibit the proliferative, colony forming, and invasion of CC, while overexpressing SCIRT of lncRNA SCIRT could promote the proliferation and invasion of CC. Western blotting analysis showed that SCIRT knockdown significantly promoted the expression of e-cadherin and inhibited the expression of n-cadherin, vimentin, MMP-9, and MMP-2, while overexpressing SCIRT of lncRNA SCIRT had the opposite effect. Besides, SCIRT knockdown could significantly inhibit the proliferative ability of CC in nude mouse. Therefore, lncRNA SCIRT may be a potential therapeutic target or a new biological diagnostic target for CC patients.

Many lncRNAs are deemed as dysregulated in CC, participating in gene regulation. For example, they can weaken or enhance the expression of target genes in the carcinogenesis process at the transcriptional level to block or promote the development of cancer. Moreover, they are closely linked to tumor-mass dimension, angiogenesis, FIGO stage, lymph node metastases, and poor prognosis. Hence, the lncRNA expression is an important prognostic indicator for CC diagnosis as well as a possible drug-target for CC therapy [21–23]. For example, 1,056 lncRNAs expressed in human cervix were reported for the first time by constructing a long-sequence gene expression library in non-tumor and intraepithelial cervical neoplasia specimens, indicating that such non-fungible lncRNA transcripts provide for driving force of cervical precursor-lesions [24]. MEG3 expression in CC is significantly lowered and this correlated with tumor-mass dimension, late FIGO stage, lymph node metastases, and positive HR⁃HPV. Meanwhile, upregulated MEG3 expression could regulate CC proliferative property and induce apoptosis, suggesting that MEG3 has a tumor suppressive effect in CC [25]. The present study first found that lncRNA SCIRT was highly expressed in CC tissues and cells, and that it was significantly correlated with the clinicopathological characteristics of patients, such as FIGO stage, tumor dimension and lymph node metastasis. Further cell functional experiments demonstrated that SCIRT knockdown could significantly inhibit the proliferation, colony formation and invasion of CC, while overexpressing SCIRT could facilitate the proliferative and invasive property of CC.

The primary etiology for CC-linked mortality is treatment failure, recurrence, and metastasis of tumors. Epithelial-to-mesenchymal transition (EMT) is a key process to induce distant metastasis of tumor [26]. For example, lncRNA UCA1 expression in CC is increased and linked to patient survival rate, and EMT-related protein expression in CC is regulated through targeting miR-155 (upregulating e-cadherin while downregulating vimentin expression) [27]. In addition, LINC00319 is highly expressed in CC tissues and cells, and overexpressing SCIRT of LINC00319 can promote cell migratory, invasive, and EMT processes in CC [28]. The results of the present study further proved that lncRNA SCIRT regulates the invasive and migratory properties of CC by affecting the expression of EMT-associated proteins, such as e-cadherin, n-cadherin, and vimentin. Besides, matrix metalloproteinases (MMPs), especially MMP-2 and MMP-9, are highly expressed in a variety of tumor tissues and play an important role in the development of tumors [29, 30]. Recently, studies have shown that MMP-2 and MMP-9 are overexpressed in CC and precancerous lesions and have certain diagnostic and prognostic value for CC [29, 31]. Our Western blotting results showed that lncRNA SCIRT knockdown markedly reduced the expression of n-cadherin, vimentin, MMP-9, and MMP-2, while lncRNA SCIRT overexpressing SCIRT had the opposite effect. To further determine the role of lncRNA SCIRT, tumorigenic experiments in nude mouse was conducted. The results demonstrated that lncRNA SCIRT knockdown could significantly inhibit the proliferation of CC in vivo.

In conclusion, the results of this study proved for the first time that lncRNA SCIRT is highly expressed in CC tissues and cells. SCIRT knockdown markedly reduced the proliferation, colony formation, and invasion of CC by promoting the expression of e-cadherin and inhibiting the expression of n-cadherin, vimentin, MMP-9, and MMP-2, while overexpressing SCIRT had the opposite effect. Besides, SCIRT knockdown could significantly inhibit the proliferative property of CC in nude mouse. Therefore, lncRNA SCIRT could be a potential therapeutic target or a new biological diagnostic target for CC patients.

## Figures and Tables

**Table 1:** Associations of SCIRT expression and clinicopathological features

Characteristics	Cases (*N* = 34)	*SCIRT expression*		*Pvalue*
		Low	High	
*Age*				
>55 years	19	9	10	0.7379
≤55 years	15	6	9	

*FIGO grade*				
I-II	14	10	4	**0.0135**
III-IV	20	5	15	

*Distal metastasis*				
Yes	18	7	11	0.7303
No	16	8	8	

*Tumor dimension*				
≤4 cm	14	11	3	**0.0013**
>4 cm	20	4	16	

*Lymph node metastasis*				
Yes	13	2	11	**0.0128**
No	21	13	8	
HPV				
HPV16+	10	5	5	0.7176
HPV18+	24	10	14	

^*∗*^means statistically significant.

## Data Availability

Emails could be sent to the address below to obtain the shared data:13770046036@163.com.
